# Supplementary material for: DNA-Binding One Finger Transcription Factor PhDof28 Regulates Petal Size in Petunia
Source: Int J Mol Sci. 2023 Jul 26;24(15):11999. doi: 10.3390/ijms241511999 (PMC10418906; doi:10.3390/ijms241511999)
Supplement: Supplementary file 1 [file ijms-24-11999-s001.zip › ijms-2490495-supplementary.pdf]

SUPPLEMENTARY MATERIALS

Supplementary figure legends

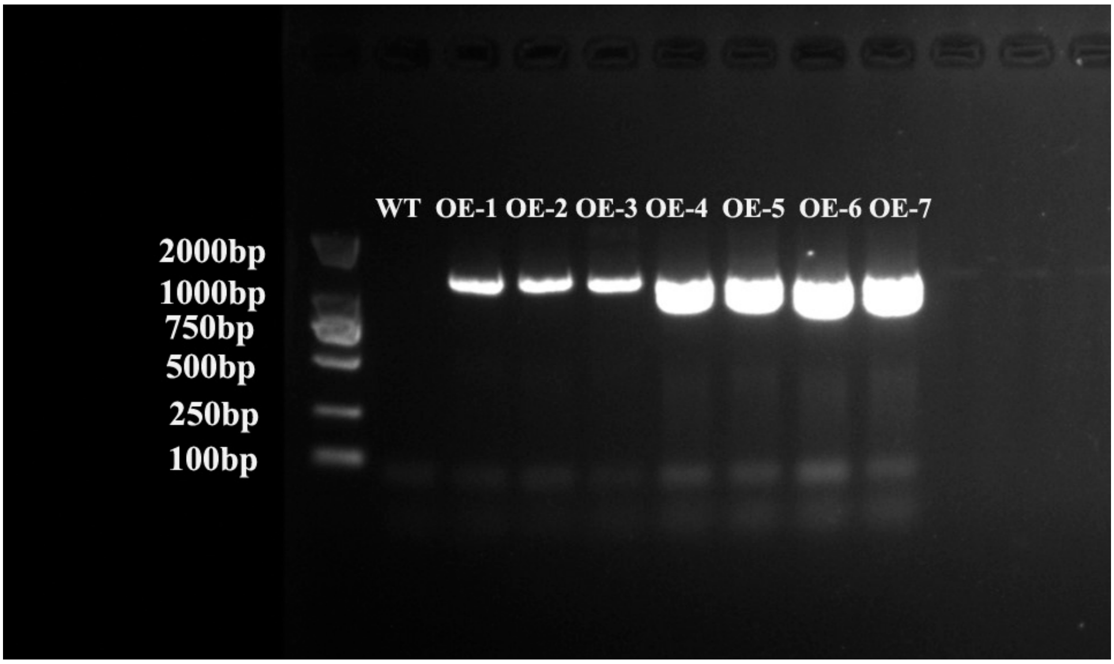

Figure S1. PCR positive detection of F_0 generation transgenic petunia.

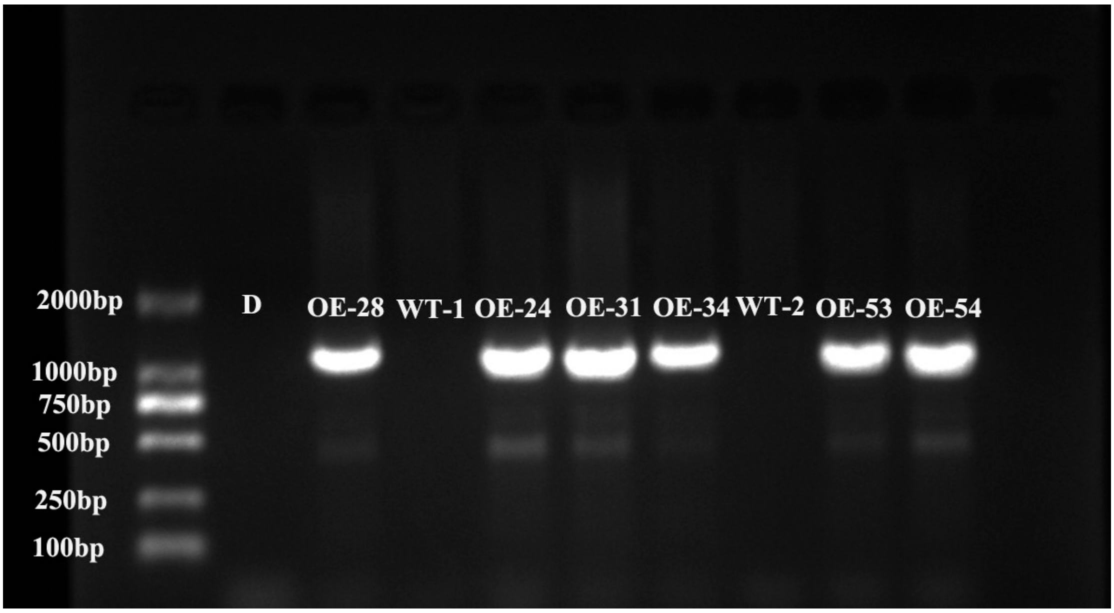

Figure S2. PCR positive detection of F_0 generation transgenic tobacco. Label D means the ddH_2O was used as the template of blank control. Label D means the ddH_2O was used as the template of blank control.

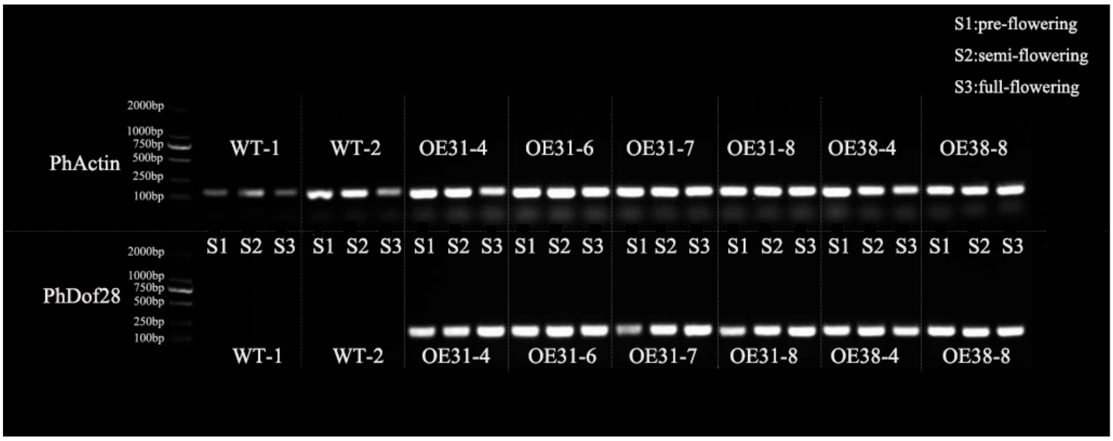

Figure S3. Semi quantitative PCR of transgenic tobacco F_1 plants.

Table S1. Primer sequence used in this study.

Primer Name	Primer Sequence
35S-F	ACGCACAATCCCACTATCCTTC
PhDof28-R	AAAAAGGAGAGATATAGATCAAGGTAG
28qpcr-F	GGGAACTTTACAAATCTGATGACG
28qpcr-R	GAAACCCGAACTATTACCACCC
PhActin-F	GTTGGACTCTGGTGATGGTGTG
PhActin-R	CCGTTCAGCAGTGGTGGTG
q-PhEXPA1-F	AGCTCCTACACTTCTCTTCCA
q-PhEXPA1-R	GGAAAAGTAAGGTTCTTATTATGAGGT
q-PhEXPA2-F	TGTTTTATGATTTCTTCCCTCTTAAA
q-PhEXPA2-R	GCATATTACTATTCCTATTCCGAATGT
q-PhEXPA3-F	GATTTTGAGGGTATTGCTATTAGATTGT
q-PhEXPA3-R	CCACCTCTGCTTACTCAGGC
q-PhBPEp-F	GGAAATGGTGACCTTGCTGG
q-PhBPEp-R	TTCCAAATTCACATCCCTCCTC
q-PhNAC100-F	AGTGCAGGAGGGAAGAAAATACA
q-PhNAC100-R	GGAGAAGCAGGTCACATGAGAATA
q-PhCesA6-F	TACAGGGTGACGAGGAAGAAGAT
q-PhCesA6-R	GCTCAAAAGCATAACACTGGAAGT
q-RhCesA9-F	AAAGGGAGCCCCAGAGTTGA
q-RhCesA9-R	CAGAATATGCAGCCTCGGAAA
q-RhCesA5-F	CCAAGGTTCGGGTCCAGTT
q-RhCesA5-R	GGATGCTGATGACTCCGTATGA
q-PhPIP1;1-F	GTCTCCTCTTTTCAGCCATTTTG
q-PhPIP1;1-R	CGCCGATCTCATTCATTCTTC
q-PhPIP2;1-F	CTAAACCACATCCTCACAACCAG
q-PhPIP2;1-R	CCACATACGTTCCTCTTACACACAT
q-XTH6-F	CAAGTTTAGAACCTGCGATACCC
q-XTH6-R	TGGTGGCTGATTTTCCTCTTC
q-XTH22-F	GGTATGGAATGGGCAAAGGT
q-XTH22-R	ATTTCTCCAGGCAGGGGTC
q-XTH23-F	GCCTTGGCATTTTCATTTTCA
q-XTH23-R	CGTTATCCGTGTCCGAGTTCAT
